# Transcriptome and proteomics conjoint analysis reveal anti‐alcoholic liver injury effect of Dianhong Black Tea volatile substances

**DOI:** 10.1002/fsn3.3763

**Published:** 2023-10-16

**Authors:** Tinghui Gao, JiaoJiao Fu, Lin Liu, Jing Bai, Yangjun Lv, Yuejin Zhu, Yu Lan, Xiaonian Cao, Huafang Feng, Caihong Shen, Sijing Liu, Shikang Zhang, Jinlin Guo

**Affiliations:** ^1^ Key Laboratory of Characteristic Chinese Medicine Resources in Southwest China, College of Pharmacy Chengdu University of Traditional Chinese Medicine Chengdu P.R. China; ^2^ College of Medical Technology Chengdu University of Traditional Chinese Medicine Chengdu P.R. China; ^3^ Hangzhou Tea Research Institute, China Coop Hangzhou P.R. China; ^4^ Luzhou Laojiao Group Co. Ltd. Luzhou P.R. China; ^5^ National Engineering Research Center of Solid‐State Brewing Luzhou P.R. China

**Keywords:** alcoholic liver injury, black tea, proteomics, transcriptome, volatile substances

## Abstract

Dianhong Black Tea, a fermented tea containing various bioactive ingredients, has been found to have a significant role in alleviating alcoholic liver injury (ALI). One of its main unique components, Dianhong Black Tea volatile substances (DBTVS), may have potential anti‐ALI effects. However, its effects and underlying molecular mechanisms are still unknown. In this study, we aimed to investigate the potential of DBTVS as an anti‐ALI agent using alcohol‐fed rats. We assessed the effect of DBTVS on ALI by analyzing serum transaminase and lipid levels, as well as conducting hematoxylin–eosin and oil red O staining. Additionally, GC‐MS was used to detect the components of DBTVS, while transcriptome, proteomics analysis, Western blot, and molecular docking were employed to uncover the underlying mechanisms. Our results demonstrated that DBTVS significantly reduced serum ALT and AST levels and improved lipid metabolism disorders. Moreover, we identified 14 components in DBTVS, with five of them exhibiting strong binding affinity with key proteins. These findings suggested that DBTVS could be a promising agent for the prevention and treatment of ALI. Its potential therapeutic effects may be attributed to its ability to regulate lipid metabolism through the PPAR signaling pathway.

## Graphical abstract

Dianhong Black Tea volatile substances may have anti‐ALI potential. Dianhong Black Tea volatile substances reduces serum ALT/AST and improves lipid metabolism. PPAR signaling pathway is the most enriched pathway affected. Dianhong Black Tea volatile substances inhibits key enzymes in PPAR pathway.

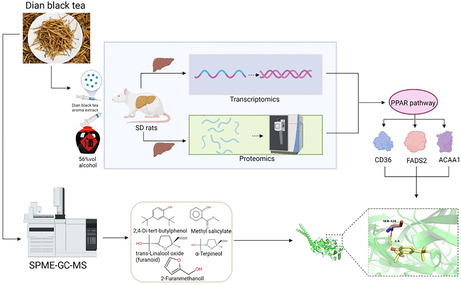

## 1 INTRODUCTION

With the progress of economic development and the rise in stress levels, alcohol consumption has become increasingly prevalent and popular. However, it is important to note that excessive alcohol intake is a significant contributor to global mortality, accounting for approximately 4% of deaths worldwide (Ayares et al., 2022). This makes it one of the major risk factors for the burden of disease on a global scale. Excessive alcohol consumption often leads to the development of alcoholic liver injury (ALI), which initially presents as alcoholic fatty liver disease and can progress to more severe conditions such as alcoholic hepatitis, liver fibrosis, cirrhosis, and even liver cancer (Addolorato et al., 2016). In fact, it is estimated that alcohol is responsible for 30% of mortality in patients with primary liver cancer globally (Akinyemiju et al., 2017). Despite numerous studies investigating treatment strategies for ALI, a definitive treatment without side effects has yet to be identified. For example, previous studies have indicated that liver regeneration is a crucial target for alleviating ALI. However, a clear treatment strategy is yet to be established (Louvet & Mathurin, 2015). While corticosteroids have shown positive effects on early ALI, it is worth noting that some patients with liver disease exhibit reduced sensitivity to corticosteroids (Louvet & Mathurin, 2015). Additionally, although TNF antagonists have the potential to improve liver function, certain studies have also reported higher rates of infection and mortality associated with TNF therapy (Louvet & Mathurin, 2015). Consequently, there is an urgent need for the development of novel and more practical treatment options.

Growing evidences the use of nutritional supplementation as an effective strategy to improve ALI (Cao et al., 2020; Guo, Cao, et al., 2022; Lai et al., 2022; Zhang et al., 2021). Tea, a widely consumed beverage worldwide, has been classified into 6 types based on production processes, namely green tea, white tea, yellow tea, oolong tea, dark tea, and black tea. These teas are known for their rich content of phenols, flavonoids, polysaccharide, and phenylpropanoid glycoside, which have been reported to possess multiple beneficial effects, including antioxidative, anti‐inflammation, as well as preventing ALI (Cao et al., 2020; Lai et al., 2022; Li, Liu, et al., 2021; Li, Mao, et al., 2021). Black tea, in particular, undergoes fermentation, resulting in a distinct aroma. Undoubtedly, such as a reduction in tea polyphenols. However, despite this reduction, black tea still exhibits a strong antioxidant capacity and demonstrates a preventive effect against ALI (Kaviarasan et al., 2008; Zhang et al., 2021). Therefore, black tea may prevent and control ALI through a mechanism that differs from that of green tea (Cao et al., 2020; Lai et al., 2022; Li, Liu, et al., 2021; Li, Mao, et al., 2021).

Aroma plays a significant role in the appeal of black tea, making it a popular choice among consumers. However, previous studies on black tea have primarily focused on its water extracts, neglecting the investigation of volatile substances and their potential anti‐ALI effects and underlying mechanisms. The chemical composition of black tea varies considerably, offering diverse health benefits. Two commonly consumed black tea types in China, namely Keemun black tea (KBT) and Dian Hong tea (DBT), possess distinct aromatic profiles. KBT exhibits a gentle aroma with a subtle rose fragrance dominated by geraniol, while DBT boasts a robust aroma with a rich floral and fruity scent primarily attributed to linalool, accompanied by a sweet fragrance. Recent research has demonstrated that DBT outperforms KBT in preventing excessive liver fat accumulation (Liao et al., 2022). Based on this finding, we hypothesized that DBT may exhibit superior anti‐ALI efficacy. Consequently, this study aims to investigate the effectiveness and mechanisms of DBT in preventing ALI.

Curcumin, a polyphenol isolated from *Curcuma longa* L. in 1870, exhibits strong antioxidant and anti‐inflammatory effects (Menon & Sudheer, 2007). It possesses hepatoprotective properties. Numerous studies have extensively examined its impact on acute and/or chronic alcohol‐induced (Li et al., 2023; Wang et al., 2019), aflatoxin‐induced (Li, Liu, et al., 2021; Li, Mao, et al., 2021), oxaliplatin‐induced (Lu et al., 2020), ischemia–reperfusion‐induced liver injury (Mokhtari‐Zaer et al., 2018), and lipopolysaccharide‐induced liver failure (Ganji et al., 2021) in experimental animal models. Therefore, curcumin was utilized as a positive control in our study.

To analyze developmental processes, gene function, adaptive and physiological stress responses at the transcriptomic and proteomic levels, high‐throughput technologies have been employed. The integration of multi‐omics analysis aids in exploring the underlying mechanism. Hence, in this study, we aimed to investigate the effect of Dianhong Black Tea volatile substances (DBTVS) on ALI and explore its potential mechanism using transcriptomics, proteomics, western blot, and molecular docking techniques.

## 2 MATERIALS AND METHODS

### 2.1 Materials

The curcumin was purchased from Shanghai Yien Chemical Technology (Shanghai, China). Rabbit polyclonal antibodies specific to CD36 (ab252922), HMGCS2 (ab137043), and ACSL1 (ab177958) were the production of Abcam (UK). CYP7A1 (DF2612), FADS2 (DF15514), and ACAA1 (DF12345) were purchased from Affinity (USA). The GAPDH (T0004) was the production of Zen Bioscience (Chengdu, China).

### 2.2 Preparation of DBTVS

DBTVS were provided by the Hangzhou Tea Research Institute (Hangzhou, China), and the DBTVS extraction was performed according to a previous study (Wang et al., 2022). Briefly, 1 kg Dian Hong Black Tea was ground and immersed in 80 L reverses osmosis water for 4 h (5°C), followed by evaporation in a condensate recovery vessel at 120°C. The released water vapor was cooled by low temperature recycling water (5°C), and then concentrated to 40% of solid content. The DBTVS were further enriched by salting‐out re‐distillation method. The DBTVS were made in the same way as the added ingredients in the Mingniang production of Luzhou Laojiao Group Co. Ltd.

### 2.3 Animal experiments

Fifty SPF male SD rats (180 ± 10 g, 6‐week‐old) were purchased from 0 (license No: SCXK (chuan) 2020‐030). Rats were housed in a temperature (22 ± 2°C) and humidity (55 ± 15%) controlled room with a 12 h light/dark cycle. All animal procedures were approved by the Ethics Committee on the Care and Use of Laboratory Animals in Chengdu University of Traditional Chinese Medicine (approving no: 2022DL‐017). After 7 days of acclimatization, all rats were randomly divided into five different groups, namely, the control group (Control, sterile water by gavage), the model group (Model, 56% [v/v] alcohol by gavage), the positive drug control group (Positive, 75 mg/kg curcumin by gavage), low‐dose DBTVS treated group (DBTVS‐L, 0.45 mL/kg DBTVS by gavage), and high‐dose DBTVS treated group (DBTVS‐H, 9 mL/kg DBTVS by gavage). All rats were given saline, curcumin, low or high doses of DBTVS once a day in the morning for 4 consecutive days. On the 5th day, rats were given saline, curcumin or DBTVS orally, except for the control group, 4 h after oral administration of 56% (v/v) alcohol (10 mL/kg; Wang et al., 2022). All rats were daily administered for 9 consecutive days. All animals had free access to food and water during the experimental period. The body weight of each rat was measured daily. At the end of experiment, the abdominal aortic blood and liver samples were collected and stored at −80°C for subsequent experiments.

### 2.4 Analysis of serum transaminase and lipid level

The serum alanine aminotransferase (ALT), aspartate aminotransferase (AST), total triglyceride (TG), total cholesterol (TC), high‐density lipoprotein cholesterol (HDL‐C), and low‐density lipoprotein cholesterol (LDL‐C) were determined by automatic biochemical analyzer (Mindry, Shenzhen, China). All protocols were followed the manufacturer recommendations.

### 2.5 HE staining and oil red O staining

The liver samples were fixed in 4% paraformaldehyde, embedded, and sectioned. Sections (5 μm) were stained with HE staining and oil red O staining according to standard procedures.

### 2.6 RNA‐sequencing and differentially expressed genes analysis

The total RNA of the liver tissues was extracted by TRIzol reagent (Beijing CoWin Biotech, China). The concentration and purity were measured using NanoDrop 2000 (Thermo Fisher Scientific, Wilmington, USA). The RNA integrity was assessed using the RNA Nano 6000 Assay Kit of the Agilent Bioanalyzer 2100 system (Agilent Technologies, CA, USA). Then, the sequencing libraries were generated using NEBNext UltraTM RNA Library Prep Kit for Illumina (NEB, USA) following manufacturer's recommendations, and index codes were added to attribute sequences to each sample. The PCR products were purified, and the library quality was assessed on the Agilent Bioanalyzer 2100 system. Finally, an Illumina platform (Illumina NovaSeq 6000 platform, San Diego) was used for to generate 15 bp paired‐end reads. For the samples with biological replicates, differential expression analysis of two groups was performed using the edgeR. The resulting *p* values were adjusted using the Benjamini and Hochberg's approach for controlling the false discovery rate. Genes with an adjusted *p* < .01 found by edgeR were assigned as differentially expressed. For the samples without biological replicates, differential expression analysis of two samples was performed using the edgeR. The *p* < .01 and |fold change| >2 were set as the threshold for significantly differential expression. Gene ontology (GO) enrichment analysis of the differentially expressed genes (DEGs) was implemented by the GOseq R packages based Wallenius noncentral hypergeometric distribution.

### 2.7 Proteome LC–MS–MS analysis

The total protein of liver samples was extracted, and the protein concentration was determined using BCA kit (Beyotime Biotechnology, China). The same amount of protein of each sample was digested by trypsin. The digested peptides were desalted with Strata X C18 (Phenomenex, USA) and then freeze‐dried in vacuum. The peptides were dissolved with 0.5 M TEAB and labeled according to the TMT kit operating instructions. The peptides were fractionated by high‐pH reverse‐phase HPLC on an Agilent 300 Extend C18 column (250 mm × 4.6 mm × 5 μm). The peptides were separated and analyzed by liquid chromatogra‐mass spectrometry (Easy‐nLC‐1200‐Orbitrap Exploris™ 480, Germany). The secondary mass spectrometry data were retrieved using Proteome Discoverer (V2.4.1.15).

### 2.8 Western blot

The liver tissues were collected for protein extraction as described previously (Liu et al., 2021). In brief, total protein was extracted from the liver tissue using RIPA protein extraction solution (Boster Biological Technology, China) supplemented with protease inhibitors (Boster Biological Technology, China). The supernatant was centrifuged at 12,000 × *g* for 15 min. The protein concentration was measured and adjusted by BCA Protein Assay kit (Beyotime Biotechnology, China). Equal amounts of protein samples (10 μg) from the liver homogenates were separated by 10% SDS‐PAGE and transferred to a polyvinylidene fluoride membranes (Millipore, USA). After blocking with 5% skimmed milk for 1 h, the membrane was incubated overnight with the flowing antibodies at 4°C including anti‐CD36 (1:1000), anti‐HMGCS2 (1:1000), anti‐ACSL1 (1:1000), anti‐FADS2 (1:500), anti‐ACAA1 (1:500), anti‐CYP7A1 (1:500), and anti‐GAPDH (1:8000). After washing with TBST for five times, these membranes were incubated with goat anti‐rabbit horseradish peroxidase for 1 h at room temperature. Positive bands were detected by enhanced chemiluminescence detection system (Bio‐Rad Laboratories, USA). Image J software was used to quantify the immunoblots.

### 2.9 The components analysis of DBTVS

The components of DBTVS were performed by headspace solid‐phase microextraction coupled with gas chromatography–mass spectrometry (SPME‐GC‐MS, Kyoto, Japan). DBTVS was transferred to a 20 mL headspace vial, and 1 g NaCl was added, while solid‐phase microextraction (Supelco, USA) was performed at 60°C after headspace injection. After thermal desorption at 250°C for 3 min, the DBTVS was tested on the chromatographic column (Agilent, J&W DB‐5MS, USA). The high purity helium carrier gas was set at constant flow of 2.5 mL/min. The temperature program was set as follows: the initial temperature of column was set to 40°C, holding for 2 min, increased by 6°C/min to 195°C, and then increased by 15°C/min to 280°C, maintaining at 280°C for 2 min. The mass spectrometer was operated in electron impact (EI) ionization mode, ion source temperature was 220°C, the interface temperature was 280°C, and scanning *m/z* 33–500.

### 2.10 Molecular docking verification

To further validate the DBTVS against ALI, molecular docking was performed for 14 chemical constituents and the 3 key target proteins. The 3D crystal structures of key target proteins (CD36 [Uniport ID: Q07969], FADS2 [Uniport ID: Q9Z122]) were obtained from the Uniport database, the ACAA1 (PDB ID: 2IIK) were obtained from the Protein Data Bank. The 3D structures of 14 chemical constituents were obtained from the PubChem. Maestro was used to predict the binding patterns for 14 compounds and three key target proteins. The docking workflow followed the “induced fit” protocol, which allows the side chains of the receptor pocket to move according to the ligand conformations, with a constraint on their positions. The target protein had good binding activity with the compounds (the docking score < −6). The molecular docking conformations was analyzed and visualized by the Pymol software (version 2.0.1).

### 2.11 Statistical analysis

GraphPad Prism 9.0 (GraphPad Software, CA, USA) was used for statistical analysis. All data were expressed as mean ± SD. One‐way analysis of variance (ANOVA), Dunnett's multiple comparisons test, and Kruskal–Wallis test were used for comparison among multiple groups. Differences between groups were considered significant at *p* < .05.

## 3 RESULTS

### 3.1 DBTVS alleviated hepatic injury in alcohol‐fed rats

**FIGURE 1:**
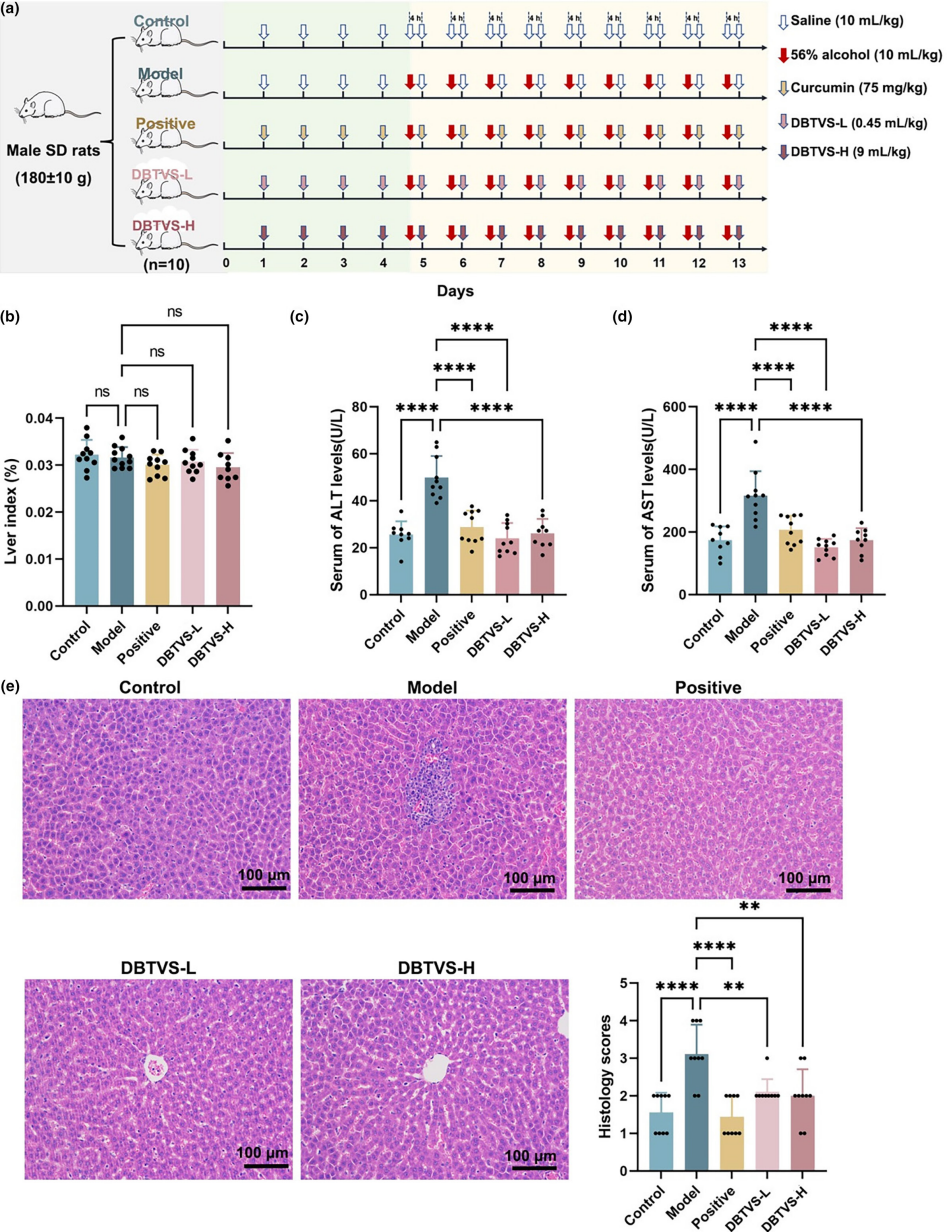
DBTVS alleviated hepatic injury in alcohol‐fed rats. (a) Schema showing the animal groups and treatments. This animal model included the pair‐fed control group (Control), the ethanol group (Model), the positive control group (Positive), and the low and high dose of DBTVS treatment groups (DBTVS‐L and DBTVS‐H). There were 10 rats in each group. (b) Liver index. (c) Serum ALT concentrations. (d) Serum AST concentrations. (h) Representative sections of liver with HE staining and quantification. Scale bar, 100 μm. ×200 magnification. Data were presented as mean ± SD. Multiple groups were tested by one‐way ANOVA followed by Dunnett‐*t* test for all statistical analyses. **p < .01 and ****p < .0001, versus Model group. ns, not statistically significant.

To evaluate the effectiveness of DBTVS in reducing liver injury, we examined the key markers of liver injury (serum AST and ALT) and histopathological changes in the livers of SD rats after DBTVS treatment (Figure 1a). Despite no significant difference in the liver index among the different groups of rats (Figure 1b), DBTVS‐L and DBTVS‐H significantly decreased serum ALT and AST levels. As depicted in Figure 1c, DBTVS‐L and DBTVS‐H reduced serum ALT levels by 51.99% and 47.73%, respectively (*p* < .0001). Similarly, DBTVS‐L and DBTVS‐H led to a significant reduction in serum AST levels by 52.19% and 44.99%, respectively (Figure 1d, *p* < .0001). Additionally, we conducted histopathological analysis of the liver using HE staining. As anticipated, DBTVS alleviated ethanol‐induced focal cell necrosis, accompanied by mild inflammatory cell infiltration and erythrocyte spillage (Figure 1e, *p* < .01). In conclusion, these findings demonstrated that DBTVS effectively mitigated liver damage in alcohol‐fed rats.

### 3.2 DBTVS improved lipid metabolism disorder in alcohol‐fed rats

**FIGURE 2:**
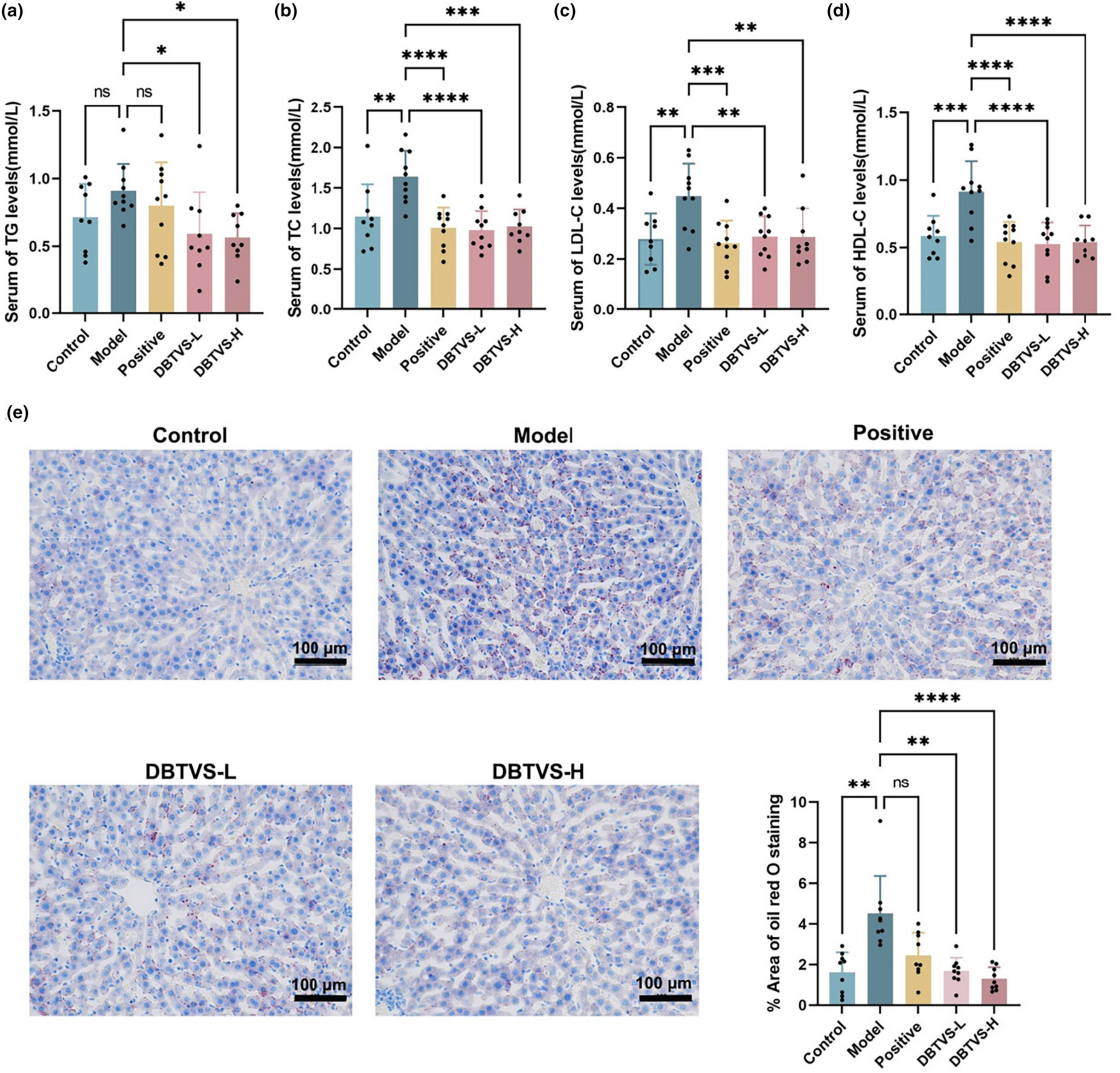
DBTVS improved lipid metabolism disorder in alcohol‐fed rats. (a) Serum TG concentrations. (b) Serum TC concentrations. (c) Serum LDL‐C concentrations. (d) Serum HDL‐C concentrations. (e) Representative sections of liver with oil red O staining and quantification. Scale bar, 100 μm. ×200 magnification. Data were presented as mean ± SD. Multiple groups were tested by one‐way ANOVA followed by Dunnett‐*t* test for all statistical analyses. *p < .05, **p < .01, and ****p < .0001, versus Model group. ns, not statistically significant.

We subsequently evaluated the impact of DBTVS on lipid metabolism in alcohol‐fed rats. In Figure 2a, it can be observed that both DBTVS‐L and DBTVS‐H significantly inhibited the increase in serum TG levels compared with the Model group (*p* < .05). Additionally, treatment with DBTVS‐L and DBTVS‐H resulted in a significant reduction in the serum TC levels, as well as the LDL‐C and HDL‐C levels (Figure 2b–d, *p* < .01). Moreover, the rats treated with DBTVS‐L and DBTVS‐H exhibited fewer hepatic lipid droplets compared to the Model rats (Figure 2e, *p* < .01). In conclusion, these findings indicated that DBTVS had a significant positive effect on correcting the disorder of lipid metabolism in alcohol‐induced mice.

### 3.3 DBTVS changed the transcriptomic and proteomics profiling of alcohol‐fed rats

**FIGURE 3:**
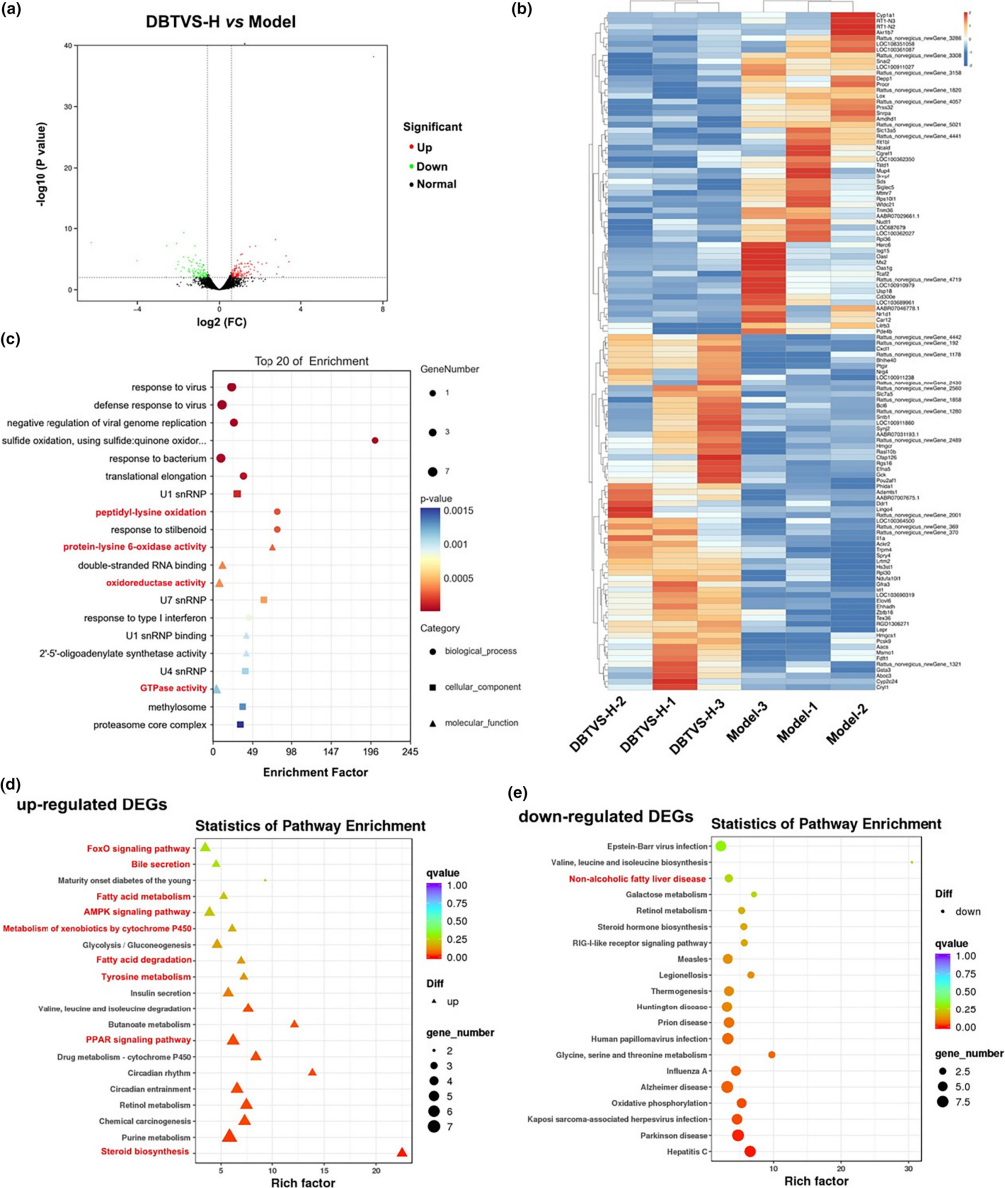
General analysis of DEGs in different groups. (a) The volcano map of upregulated and downregulated DEGs between different groups. (b) Cluster analysis of DEGs between different groups. The top 70 DEGs were shown in the heat map as red indicates a highly expressed gene, and blue indicates a low expressed gene. (c) GO analysis of DEGs. (d, e) Up‐regulated (d) and down‐regulated (e) analysis of DEGs.

**FIGURE 4:**
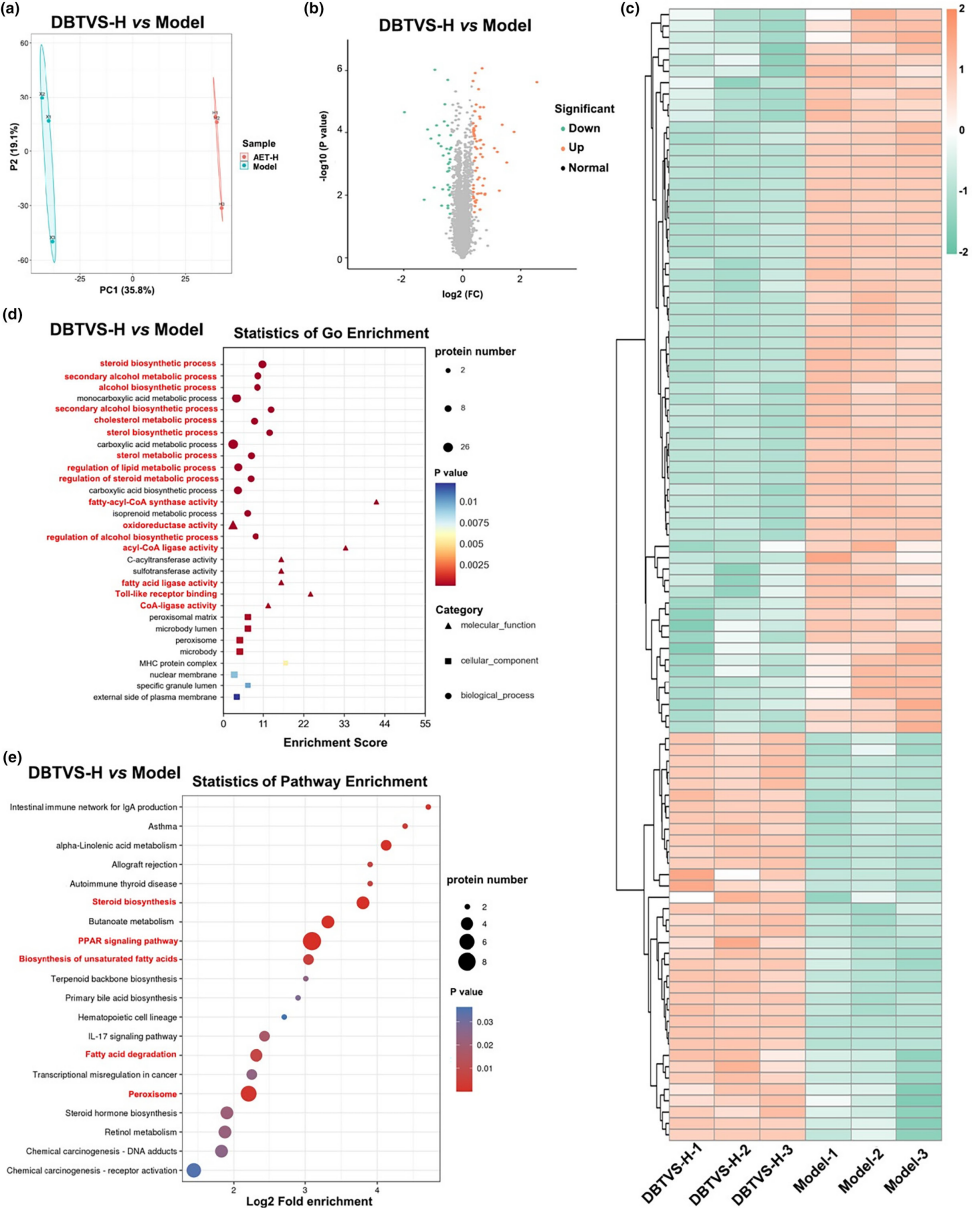
General analysis of DAPs in different groups. (a) Principal component analysis of DAPs among different groups. (b)The volcano map of upregulated and downregulated DAPs between different groups. (c) Cluster analysis of DAPs between different groups. The top 80 DAPs were shown in the heat map as red indicates a highly expressed protein, and green indicates a low expressed protein. (d, e) GO (d) and KEGG (e) analysis of DAPs.

**FIGURE 5:**
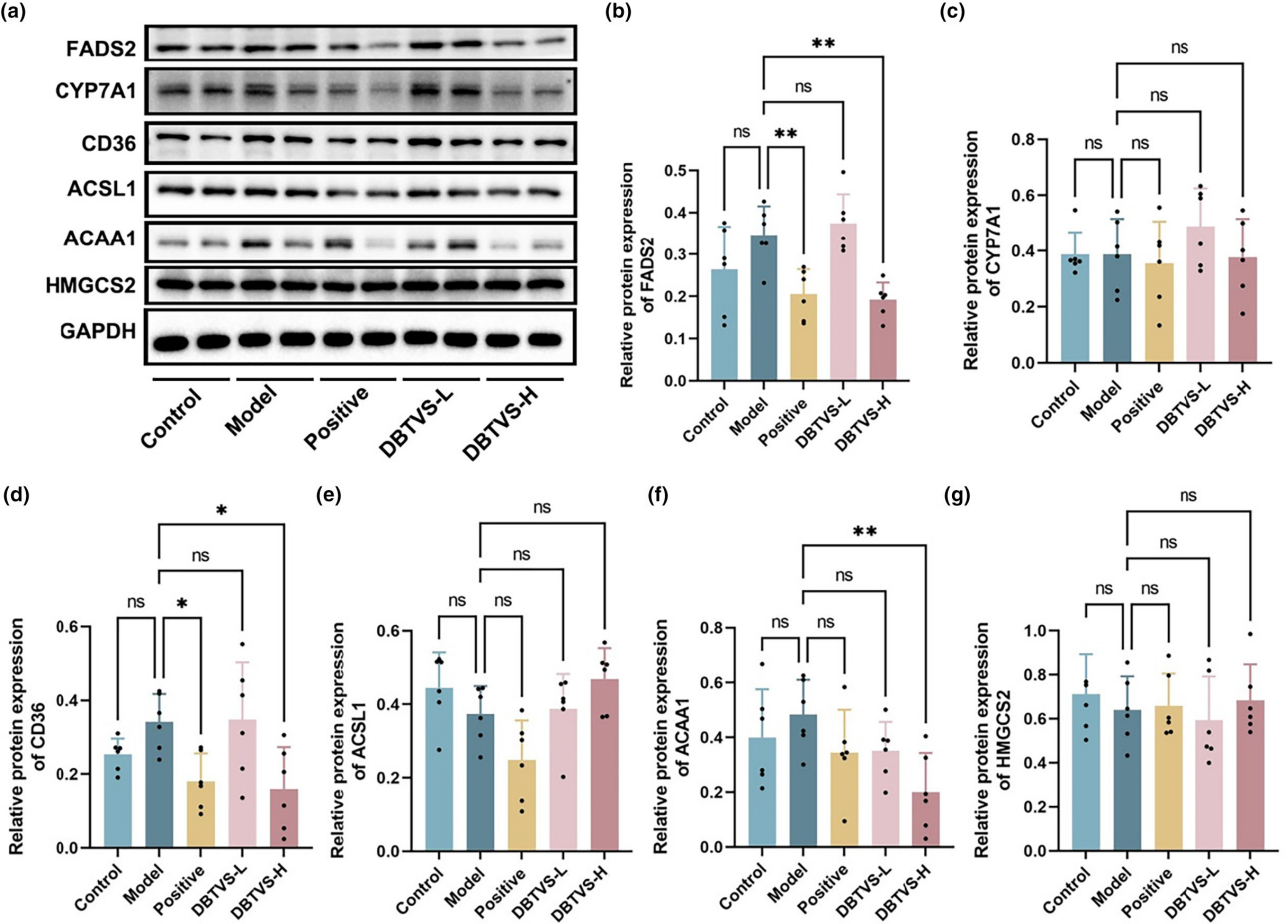
DBTVS decreased the expression of FADS2, CD36, and ACAA1. (a) Representative blots of FADS2, CYP7A1, CD36, ACSL1, ACAA1, and HMGCS2. (b) The protein expression levels of FADS2 between different groups. (c) The protein expression levels of CYP7A1 between different groups. (d) The protein expression levels of CD36 between different groups. (e) The protein expression levels of ACSL1 between different groups. (f) The protein expression levels of ACAA1 between different groups. (g) The protein expression levels of HMGCS2 between different groups. Data were presented as mean ± SD. Multiple groups were tested by one‐way ANOVA followed by Dunnett‐*t* test for all statistical analyses. *p < .05 and **p < .01, versus Model group. ns, not statistically significant.

To investigate the molecular mechanism of DBTVS on liver injury and lipid metabolism in alcohol‐fed rats, we performed hepatic transcriptome and proteome analysis (Figures 3 and 4). The DEGs between the DBTVS‐H and Model group were obtained by calculating FPKM. As shown in Figure 3a,b, we observed 149 upregulated DEGs and 123 downregulated DEGs. Additionally, the principal component analysis revealed a significant difference in protein expression between the two groups (Figure 4a). We identified 36 upregulated proteins and 64 downregulated proteins (Figure 4b,c). To understand how DBTVS affects ALI, GO and KEGG enrichment analyses were performed to further elucidate the functions and related pathways of DEGs (Figure 3c–e) and DAPs (Figure 4d,e). Among the annotated pathways, a majority were closely linked to lipid metabolism, including PPAR signaling pathway, steroid biosynthesis, fatty acid metabolism, and fatty acid degradation. In particular, two pathways, PPAR signaling pathway and steroid biosynthesis. Among them, fatty acid desaturase 2 (FADS2), cholesterol 7a‐hydroxylase (CYP7A1), cluster of differentiation 36 (CD36), long‐chain acyl‐CoA synthetase (ACSL1), acetyl coenzyme A carboxylase (ACAA1), and hydroxymethylglutaryl‐CoA synthase (HMGCS2) are important factors in regulating lipid metabolism. We further confirmed the expression of these proteins by Western blot. As shown in Figure 5, the relative protein expression levels of FASD, CD36, and ACAA1 were markedly suppressed after DBTVS‐H treatment (*p* < .05).

### 3.4 Chemical components in DBTVS and binding site analysis of key proteins

**FIGURE 6:**
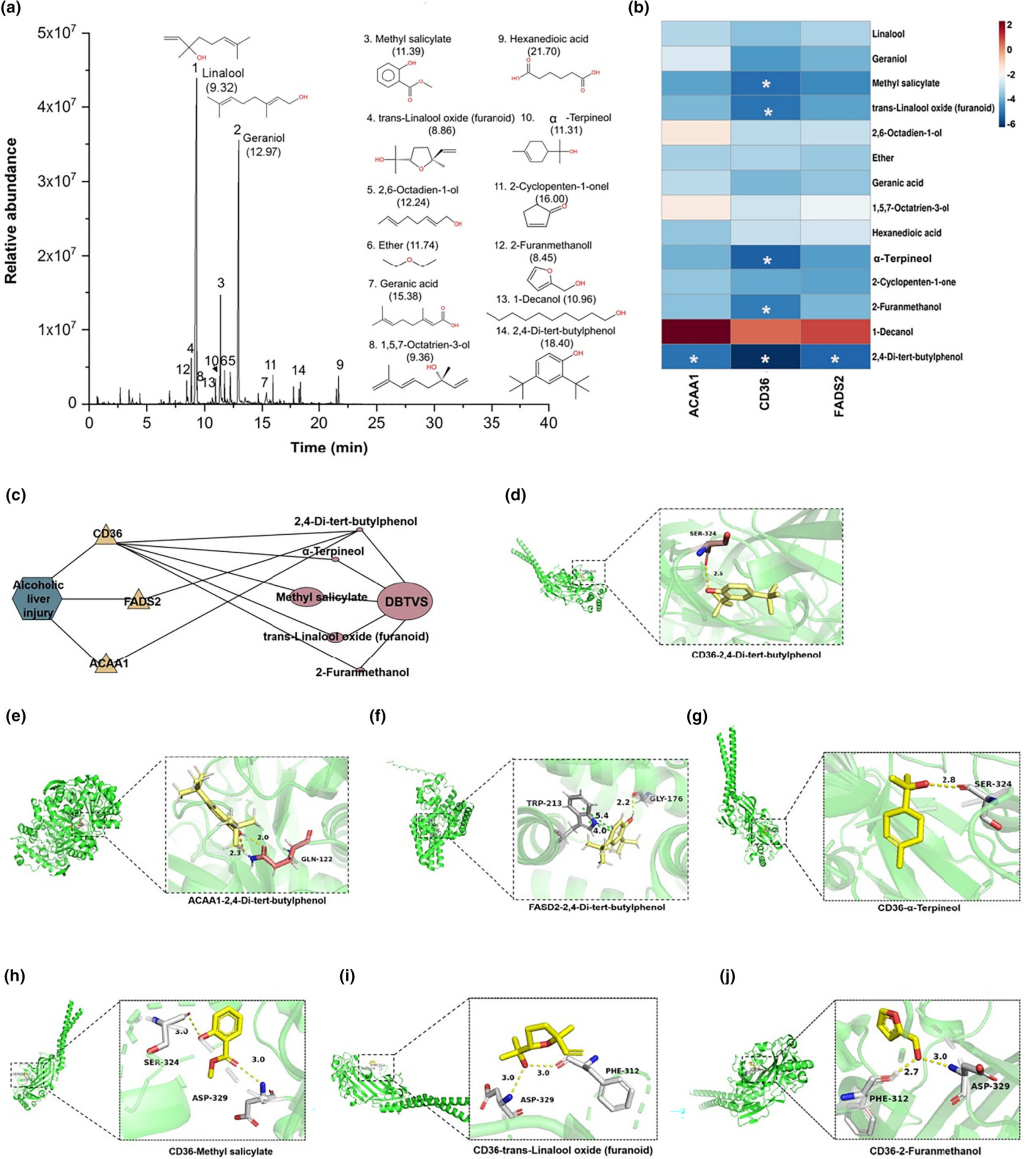
Chemical components in DBTVS and binding site analysis of key proteins. (a) Total ion chromatograms and chemical structures of DBTVS. (b) The scores of chemical constituents‐key target proteins docking were shown in heat map as red indicates a high score, and blue indicates a low score. *The score <−6. (e) The network between chemical constituents and key target proteins. (d–j) The possible binding sites of chemical constituents and key target proteins.

To investigate the potential involvement of chemical constituents of DBTVS in the regulation of inhibition of the above‐mentioned key proteins, we initially conducted GC‐MS analysis to identify the main chemical components in DBTVS. As shown in Figure 6a and Table 1 (In Table 1, the criteria for screening key volatiles is that area% greater than 1%.), 14 compounds were identified, including eight alcohols (linalool, geraniol, *trans*‐linalool oxide (furanoid), 2,6‐octadien‐1‐ol, 1,5,7‐octatrien‐3‐ol, α‐terpineol, 2‐furanmethanol, and 1‐decanol), 1 ketones (2‐cyclopenten‐1‐one), and five others (methyl salicylate, ether, geranic acid, hexanedioic acid, and 2,4‐di‐tert‐butylphenol). We then conducted protein molecular simulations of the binding modes of ACAA1, CD36, and FADS2 to the 14 compounds. As shown in Figure 6b,c and Table 2, we found that 2,4‐di‐tert‐butylphenol had the highest binding free energy with ACAA1, CD36, as well as FADS2, and the structures and binding modes of them were shown in Figure 6d,f. In addition, α‐terpineol, methyl salicylate, *trans*‐linalool oxide (furanoid), and 2‐furanmethanol exhibited the potential to bind to CD36 (Figure 6b–j). Subsequently, we performed protein molecular simulations to investigate the binding modes of ACAA1, CD36, and FADS2 with these 14 compounds. Figure 6b,c and Table 2 display the results, indicating that 2,4‐di‐tert‐butylphenol exhibited the highest binding free energy with ACAA1, CD36, and FADS2. The structures and binding modes of these interactions are presented in Figure 6d–f. Moreover, α‐terpineol, methyl salicylate, *trans*‐linalool oxide (furanoid), and 2‐furanmethanol demonstrated potential binding capabilities with CD36 (Figure 6b–j).

**TABLE 1:** GC‐MS analytical results of volatile substances of DBTVS.

Count	Peak	*t*_R_/time	Compounds	Classify	Area %
1	13	9.32	Linalool	Alcohol	41.19
2	30	12.97	Geraniol	Alcohol	26.81
3	21	11.39	Methyl salicylate	Other	5.46
4	12	8.86	*trans*‐Linalool oxide (furanoid)	Alcohol	2.80
5	28	12.24	2,6‐Octadien‐1‐ol	Alcohol	1.70
6	24	11.74	Ether	Other	1.63
7	36	15.38	Geranic acid	Other	1.49
8	14	9.36	1,5,7‐Octatrien‐3‐ol	Alcohol	1.45
9	48	21.70	Hexanedioic acid	Other	1.32
10	20	11.31	α‐Terpineol	Alcohol	1.31
11	39	15.99	2‐Cyclopenten‐1‐one	Ketones	1.26
12	10	8.45	2‐Furanmethanol	Alcohol	1.18
13	19	10.96	1‐Decanol	Alcohol	1.14
14	45	18.39	2,4‐Di‐tert‐butylphenol	Other	1.03

**TABLE 2:** Molecular docking analytical results between chemical constituents and target proteins.

Compounds	Protein	Residue	Distance	Category	Score
2,4‐Di‐tert‐butylphenol	CD36	SER324	2.6	Hydrogen bond	−7.698
	FADS2	GLY176	2.2	Hydrogen bond	
		TRP213	4.0	π‐π	−6.618
			5.4		
	ACAA1	GLN122	2.0	Hydrogen bond	−6.311
			2.3		
α‐Terpineol	CD36	SER324	2.8	Hydrogen bond	−6.741
Methyl salicylate	CD36	SER324	2.0	Polar covalent bonds	−6.384
*trans*‐Linalool oxide (furanoid)	CD36	ASP329	2.0	Hydrogen bond	−6.339
2‐Furanmethanol	CD36	ASP329	3.0	Hydrogen bond	−6.125
		PHE312	2.7		

## 4 DISCUSSION

Alcohol consumption is a very common social and cultural characteristic worldwide. However, alcohol abuse poses a huge medical burden and has become a global public health problem (Addolorato et al., 2016; Akinyemiju et al., 2017; Ayares et al., 2022). Alcohol is one of the most common contributors to liver diseases, including ALI, which poses a significant threat to human health (Addolorato et al., 2016; Akinyemiju et al., 2017; Ayares et al., 2022). Numerous studies have shown that various bioactive components in foods, such as tea, have hepatoprotective activity, with the advantages of low toxic side effects, multiple pathways, and multiple targets (Cao et al., 2020; Guo, Cao, et al., 2022; Lai et al., 2022; Zhang et al., 2021). Currently, several studies have demonstrated the potential of green tea in alleviating ALI through the activation of the PI3K/Akt/eNOS pathway, induction of Nrf2‐mediated antioxidant effect, and inhibition of NF‐κB expression (Wang et al., 2017; Zhang et al., 2021). However, limited research has been conducted on the effects of black tea, white tea, and oolong tea on ALI.

Black tea is a highly popular beverage that accounts for a significant portion of global tea consumption. Unlike green tea, which does not undergo fermentation, black tea is fully fermented. This fermentation process reduces the tea polyphenol content and gives rise to new components such as theaflavin and thearubigin. The main constituents of black tea are caffeine and flavonoids (Lai et al., 2022), which contribute to its enhanced aroma and sweet, mellow characteristics. Studies have shown that black tea exerts a sobering effect by modulating the activities of key enzymes involved in ethanol metabolism, oxidative stress, and inflammatory factors associated with liver injury. This effect may be attributed to the polyphenol oxidation products of fermented tea (Lai et al., 2022). However, volatile substances are considered the main contributor to the unique flavor of black tea, making it preferred by consumers (Ma et al., 2022). Despite this, there is limited research on the volatile substances of black tea. In a recent study, Ma et al. (2022) analyzed the aroma profile of Dian Hong Black Tea by GC‐MS and GC‐O. They found that the aroma composition of the black tea differed significantly from that of water‐extracted black tea, with alcohols being the predominant compounds rather than polyphenols and flavonoids (Ma et al., 2022). Nevertheless, the biological functions of these aromatic compounds in tea remain unclear.

In this study, we extracted volatile substances from Dian Hong black tea (DBTVS) and prepared an aqueous solution. A previous study discovered that inhaling a steam extract condensate of green tea leaves in ethanol could enhance antioxidant capacity in mice (Li et al., 2017). However, our previous research also revealed that these organic extraction methods, although they yield more volatile substances, often result in the presence of pigments and organic solvents. These components can impact the color, efficacy, and safety of the product, making it unsuitable for applications in food science. Therefore, we primarily chose the method of water extraction and distillation to prepare a colorless transparent aqueous solution that retains the characteristic aroma of the original tea while ensuring safety.

To the best of our knowledge, our study is the first to conduct in vivo experimental research using volatile water solubilizers from tea leaves. Additionally, we are the first to evaluate the anti‐ALI effects of these solubilizers in alcohol‐fed rats. Similar to previous studies, we observed a significant increase in serum transaminase levels, which are biomarkers reflecting liver function, in the alcohol‐fed rats (Cao et al., 2020; Guo, Cao, et al., 2022; Lai et al., 2022; Zhang et al., 2021). Importantly, we found that DBTVS significantly suppressed the elevation of ALT and AST levels induced by alcohol consumption and alleviated liver dysfunction (Figure 1). Alcohol consumption is known to disrupt normal hepatic lipid metabolism by promoting fatty acid synthesis while inhibiting fatty acid oxidation and transport. In our study, we observed that DBTVS significantly decreased serum TC, TG, and LDL‐C, which is consistent with the results for liver oil red O staining (Figure 2). However, it is worth noting that our study also revealed a reduction in HDL‐C levels following DBTVS administration, which contradicts the findings of some other studies (Cao et al., 2022; Guo, Zhu, et al., 2022; Lin et al., 2021). Previous beliefs considered HDL‐C as a “good cholesterol,” which seems to prevent the development of atherosclerotic disease. However, recent studies have demonstrated that the cholesterol efflux capacity of serum HDL is a more accurate and independent predictor of the risk of developing atherosclerotic cardiovascular disease than HDL‐C (Ronsein & Heinecke, 2017; Toth et al., 2014). In conclusion, our findings suggested that DBTVS had favorable effect on ALI.

To investigate the mechanisms associated with DBTVS alleviation of ALI, we further analyzed the changes in RNA and protein levels in the liver by transcriptomic and proteomic analysis. Interestingly, we found that DBTVS mainly influenced lipid metabolic pathways, particularly the PPAR signaling pathway. Previous studies have shown that alcohol consumption enhances fatty acid uptake and lipogenesis and decrease fatty acid oxidation, resulting in the accumulation of lipids in the liver (Lívero & Acco, 2016). This suggested that DBTVS may alleviate ALI by regulating lipid metabolism. Our combined transcriptome and proteomics analysis identified several lipid metabolism‐related proteins, FADS2, CD36, ACCA1, CYP7A1, ACSL1, and HMGCS2, that were inhibited by DBTVS. However, Western blot analysis only confirmed significant decreases in FADS2, CD36, and ACCA1 (Figure 5). This discrepancy may be attributed to variations in sensitivity between the two methods. FASD2, a key enzyme involved in the synthesis of long‐chain polyunsaturated fatty acids, has been associated with weight gain (Lattka et al., 2010; Santana et al., 2022). CD36 is involved in the progression of ALI by promoting triglyceride accumulation and subsequent lipid‐induced endoplasmic reticulum stress (Lebeau et al., 2019). Similarly, ACC, a rate‐limiting enzyme in the de novo adipogenesis pathway, catalyzes the carboxylation of acetyl‐CoA to malonyl‐CoA (Numa et al., 1970). Our results indicated that DBTVS may alleviate lipid deposition, reduce TG accumulation, and mitigate hepatoxicity in ALI rats by regulating proteins involved in lipid metabolism (FADS2, CD36 and ACCA1). Furthermore, transcriptomic and proteomic analysis revealed that DBTVS may also impact inflammatory signaling pathways in alcohol‐fed rat (Figure 4), as evidenced by the upregulation of MMP9 expression. Alcoholic hepatitis can lead to liver fibrosis, characterized by the deposition of collagens, elastin, and fibronectin. Considering the crucial role of MMP9 in the regulation and degradation of gelatin and collagen (Wu et al., 2022), exploring the potential anti‐inflammatory mechanisms of DBTVS in alleviating ALI warrants further investigation.

To further analyze the potential molecular mechanism, we analyzed the component of DBTVS. Compared with the study of Ma et al, we also detected linalool, geraniol, methyl salicylate, and 2,4‐di‐tert‐butylphenol in DBTVS (Ma et al., 2022). Among the 14 substances, linalool and geraniol were found to be the most abundant, with a total relative content of 68% (Table 1). This finding differs slightly from their study, which reported linalool as the highest, followed by phenethyl alcohol and phenylacetaldehyde (Ma et al., 2022). The variation in results could be attributed to differences in extraction methods. Our molecular docking results showed that linalool and geraniol exhibited low affinity to FADS2, CD36, and ACCA1 (Table 2). However, previous studies have demonstrated that linalool and geraniol can inhibit liver injury through antioxidant signaling pathways (El Azab & Abdulmalek, 2022; Hsouna et al., 2022; Miao et al., 2022). Furthermore, both compounds have broad‐spectrum antibacterial effects (Gu et al., 2022; Li, He, et al., 2022; Li, Ren, et al., 2022) and the potential to modulate the gut microbiota (Ricci et al., 2022). Recent research has emphasized the role of the gut microbiota in the development of ALI (Du et al., 2022; Lucey et al., 2009). These findings suggest that the gut microbiota could also be a potential target for DBTVS in alleviating ALI, warranting further investigation.

Furthermore, in our study, although the relative concentration of 2,4‐di‐tert‐butylphenol was low, it exhibited a strong affinity for three key proteins, suggesting its potential to act through multiple targets. Previous studies have demonstrated the anti‐inflammatory and antioxidant effects of 2,4‐di‐tert‐butylphenol (Nair et al., 2020; Vahdati et al., 2022). However, its effects on the regulation of lipid metabolism have not been investigated yet, which warrants further exploration. Our molecular docking results indicated that 2, 4‐di‐tert‐butylphenol, α‐terpineol, methyl salicylate, *trans*‐linalool oxide (furanoid), 2‐furanmethanol, and other chemical components may exert an anti‐ALI effect by interacting with CD36. Additionally, 2,4‐Di‐tert‐butylphenol can also exert an anti‐ALI effect on FADS2 and ACAA. These results suggested that the aforementioned substances may be the active components of DBTVS. However, it is important to consider that metabolites of other constituents, which are absorbed into the bloodstream after intestinal absorption, may also activate the PPAR signaling pathway or the MMP9 pathway. Therefore, we will conduct further screening of the metabolites in DBTVS through in vitro and in vivo experiments to gain more insight into the interaction between metabolites and CD36, FADS2, and ACAA.

## 5 CONCLUSION

In conclusion, the present study suggested that DBTVS treatment effectively inhibited ALI, possibly by modulating hepatic lipid metabolism. Furthermore, our finding revealed a potential biochemical mechanism of DBTVS against ALI, which could provide an important basis for the development of DBTVS as a new functional component against ALI.

## AUTHOR CONTRIBUTIONS

**tinghui Gao:** Data curation (equal); formal analysis (equal); investigation (equal); methodology (equal); writing – original draft (equal). **Jiaojiao Fu:** Data curation (equal); formal analysis (equal); investigation (equal). **Lin Liu:** Data curation (equal); investigation (equal). **Jing Bai:** Data curation (equal); investigation (equal). **Yangjun Lv:** Methodology (equal). **Yuejin Zhu:** Formal analysis (equal). **Yu Lan:** Writing – original draft (equal). **Xiaonian Cao:** Writing – original draft (equal). **Huafang Feng:** Writing – review and editing (equal). **Caihong Shen:** Conceptualization (equal); funding acquisition (equal). **sijing Liu:** Methodology (equal); writing – original draft (equal). **Shikang Zhang:** Conceptualization (equal); funding acquisition (equal); validation (equal). **Jinlin Guo:** Conceptualization (equal); funding acquisition (equal); validation (equal); writing – review and editing (equal).

## FUNDING INFORMATION

This research was financially supported by National Engineering Technology Research Center of Solid‐state Brewing, China (no. 2021ZYD0102).

## CONFLICT OF INTEREST STATEMENT

The authors declare that they have no conflict of interest.

## ETHICS APPROVAL

All animal procedures were approved by the Ethics Committee on the Care and Use of Laboratory Animals in Chengdu University of Traditional Chinese Medicine (approving no. 2022DL‐017).

## Data Availability

The authors confirm that the data supporting the findings of this study are available within the manuscript.
